# Measuring adherence to inhaled control medication in patients with asthma: Comparison among an asthma app, patient self‐report and physician assessment

**DOI:** 10.1002/clt2.12210

**Published:** 2023-02-15

**Authors:** Afonso Cachim, Ana Margarida Pereira, Rute Almeida, Rita Amaral, Magna Alves‐Correia, Pedro Vieira‐Marques, Claudia Chaves‐Loureiro, Carmelita Ribeiro, Francisca Cardia, Joana Gomes, Carmen Vidal, Eurico Silva, Sara Rocha, Diana Rocha, Maria Luís Marques, Rosália Páscoa, Daniela Morais, Ana Margarida Cruz, Marta Santalha, José Augusto Simões, Sofia da Silva, Diana Silva, Rita Gerardo, Filipa Todo Bom, Ana Morete, Inês Vieira, Pedro Vieira, Rosário Monteiro, Maria Rosário Raimundo, Luís Monteiro, Ângela Neves, Carlos Santos, Ana Margarida Penas, Rita Regadas, José Varanda Marques, Inês Rosendo, Margarida Abreu Aguiar, Sara Fernandes, Carlos Seiça Cardoso, Filipa Pimenta, Patrícia Meireles, Mariana Gonçalves, João Almeida Fonseca, Cristina Jácome

**Affiliations:** ^1^ Faculty of Medicine University of Porto Porto Portugal; ^2^ Center for Health Technology and Services Research (CINTESIS) Faculty of Medicine University of Porto Porto Portugal; ^3^ Department of Community Medicine, Information and Health Decision Sciences (MEDCIDS) Faculty of Medicine University of Porto Porto Portugal; ^4^ Allergy Unit Instituto and Hospital CUF‐Porto Porto Portugal; ^5^ Department of Cardiovascular and Respiratory Sciences Porto Health School Polytechnic Institute of Porto Porto Portugal; ^6^ Department of Women's and Children's Health Pediatric Research Uppsala University Uppsala Sweden; ^7^ Pulmonology Department Hospitais da Universidade de Coimbra Centro Hospitalar e Universitário de Coimbra Coimbra Portugal; ^8^ Clinical Academic Center of Coimbra Coimbra Portugal; ^9^ Serviço Imunoalergologia Centro Hospitalar e Universitário de Coimbra Coimbra Portugal; ^10^ Unidade de Saúde Familiar (USF) Terras de Azurara Agrupamento de Centros de Saúde Dão Lafões Mangualde Portugal; ^11^ Serviço de Imunoalergologia, Unidade I Centro Hospitalar Vila Nova de Gaia/Espinho Vila Nova de Gaia Portugal; ^12^ Servicio de Alergia Complejo Hospitalario Universitario de Santiago Santiago de Compostela Spain; ^13^ USF João Semana Agrupamento de Centros de Saúde (ACES) Baixo Vouga Ovar Portugal; ^14^ USF Arte Nova ACES Baixo Vouga Oliveirinha Portugal; ^15^ USF Sá de Miranda ACES Cávado II ‐ Gerês/Cabreira Vila Verde Portugal; ^16^ Serviço de Imunoalergologia Hospital da Senhora da Oliveira Guimarães Portugal; ^17^ USF Abel Salazar ACES Gaia Vila Nova de Gaia Portugal; ^18^ USF Corgo ACES Douro I ‐ Marão e Douro Norte Vila Real Portugal; ^19^ USF Bom Porto ACES Grande Porto V ‐ Porto Ocidental Porto Portugal; ^20^ Serviço de Pediatria Hospital da Senhora da Oliveira Guimarães Portugal; ^21^ USF Caminhos do Cértoma ACES Baixo Mondego Pampilhosa Portugal; ^22^ Department of Medical Sciences University of Beira Interior Covilhã Portugal; ^23^ USF Cuidarte Unidade Local de Saúde do Alto Minho Portuzelo Portugal; ^24^ Serviço de Imunoalergologia Centro Hospitalar Universitário de São João Porto Portugal; ^25^ Serviço de Pneumologia Hospital Santa Marta Centro Hospitalar Universitário de Lisboa Central Lisboa Portugal; ^26^ Serviço de Pneumologia Hospital Beatriz Ângelo Loures Portugal; ^27^ Serviço de Imunoalergologia Hospital Infante D Pedro Centro Hospitalar Baixo Vouga Aveiro Portugal; ^28^ Unidade de Cuidados Saúde Personalizados Arnaldo Sampaio ACES Pinhal Litoral Leiria Portugal; ^29^ USF Mondego ACES Baixo Mondego Coimbra Portugal; ^30^ USF Homem do Leme ACES Porto Ocidental Porto Portugal; ^31^ USF Marquês ACES Pinhal Litoral Pombal Portugal; ^32^ USF Esgueira+ ACES Baixo Vouga Esgueira Portugal; ^33^ USF Araceti ACES Baixo Mondego Arazede Portugal; ^34^ USF Santo António ACES Cávado III ‐ Barcelos/Esposende Barcelos Portugal; ^35^ USF Baltar ACES Tâmega II ‐ Vale do Sousa Sul Baltar Portugal; ^36^ USF Aquilino Ribeiro ACES Douro II ‐ Douro Sul Moimenta da Beira Portugal; ^37^ USF Viseu‐Cidade ACES do Dão Lafões Viseu Portugal; ^38^ USF Coimbra Centro ACES Baixo Mondego Coimbra Portugal; ^39^ USF Valongo ACES Grande Porto III ‐ Maia/Valongo Valongo Portugal; ^40^ UCSP São João da Pesqueira ACES Douro Sul São João da Pesqueira Portugal; ^41^ USF Condeixa ACES Baixo Mondego Condeixa‐a‐Nova Portugal; ^42^ UCSP Figueira da Foz Sul ACES Baixo Mondego Lavos Portugal; ^43^ USF Almedina ACES Douro II ‐ Douro Sul Lamego Portugal; ^44^ USF Antonina ACES Ave/Famalicão Requião Portugal; ^45^ MEDIDA – Medicina, Educação, Investigação, Desenvolvimento e Avaliação Porto Portugal

**Keywords:** asthma, clinical decision support systems, eHealth, medication adherence, mHealth, mobile apps, patient participation, self‐management, smartphone, technology assessment

## Abstract

**Background:**

Previous studies have demonstrated the feasibility of using an asthma app to support medication management and adherence but failed to compare with other measures currently used in clinical practice. However, in a clinical setting, any additional adherence measurement must be evaluated in the context of both the patient and physician perspectives so that it can also help improve the process of shared decision making. Thus, we aimed to compare different measures of adherence to asthma control inhalers in clinical practice, namely through an app, patient self‐report and physician assessment.

**Methods:**

This study is a secondary analysis of three prospective multicentre observational studies with patients (≥13 years old) with persistent asthma recruited from 61 primary and secondary care centres in Portugal. Patients were invited to use the InspirerMundi app and register their inhaled medication. Adherence was measured by the app as the number of doses taken divided by the number of doses scheduled each day and two time points were considered for analysis: 1‐week and 1‐month. At baseline, patients and physicians independently assessed adherence to asthma control inhalers during the previous week using a Visual Analogue Scale (VAS 0–100).

**Results:**

A total of 193 patients (72% female; median [P25–P75] age 28 [19–41] years old) were included in the analysis. Adherence measured by the app was lower (1 week: 31 [0–71]%; 1 month: 18 [0–48]%) than patient self‐report (80 [60–95]) and physician assessment (82 [51–94]) (*p* < 0.001). A negligible non‐significant correlation was found between the app and subjective measurements (*ρ* 0.118–0.156, *p* > 0.05). There was a moderate correlation between patient self‐report and physician assessment (*ρ* = 0.596, *p* < 0.001).

**Conclusions:**

Adherence measured by the app was lower than that reported by the patient or the physician. This was expected as objective measurements are commonly lower than subjective evaluations, which tend to overestimate adherence. Nevertheless, the low adherence measured by the app may also be influenced by the use of the app itself and this needs to be considered in future studies.

## 1 BACKGROUND

Asthma is a chronic inflammatory disease of the airways, characterised by wheezing, cough, and shortness of breath.^1^ The impact of asthma on the population is twofold: it greatly reduces the patients' quality of life and it is responsible for premature mortality.^1^ This impact is of great concern as asthma is the most prevalent noncommunicable disease among children,^2^ affecting as much as 10% of children, and 5% of adults in the European Union (EU).^3^ The direct and indirect costs of this disease are substantial, with an estimated 5.2 billion disability‐adjusted life years being lost within the EU every year, which translates into a cost of €72 billion.^4^

The core of effective asthma treatment relies on inhaled controller medication.^1^, ^5^ This treatment in association with proper adherence is crucial to minimise future risk of exacerbations and to achieve good symptom control, both long‐term clinical goals of asthma management.^1^, ^6^ In fact, the lack of adherence to inhaled medications is an important risk factor for exacerbations and asthma‐related death.^7^, ^8^ However, despite its fundamental role, adherence is low in both children and adults with asthma, thus compromising the efficacy of the treatment and, ultimately, hindering asthma‐related outcomes and quality of life.^7^, ^8^ Therefore, in order to improve healthcare utilisation and costs it is clear the importance of increasing inhaler adherence in patients with asthma.^9^

To objectively evaluate patients' adherence, it is first necessary to have established adherence measurements. However, to date, there is no standard method to evaluate adherence in clinical practice, with several methods being tried, including pharmacy refill, electronic monitoring, patient self‐report and physician assessment. Among these, pharmacy refills can be difficult to implement in a clinical setting as they rely on electronic prescriptions and it cannot be assumed that all dispensed medication was taken by the patient according to the treatment plan.^10^, ^11^ Electronic monitoring devices, despite being effective, are expensive, which limits their widespread use in clinical practice.^12^ In contrast, patient self‐report and physician assessment are inexpensive and easily integrated in clinical practice; however, they tend to overestimate adherence.^13^ Thus, in clinical settings, decision making regarding asthma inhaler medication is nowadays still heavily dependent on subjective measurements, with both patients and physicians tending to misjudge adherence,^14^, ^15^ and discordance occurring in half of the cases.^16^

With the purpose of improving clinical decisions arises the necessity of developing more objective measures that can at the same time be easily used in routine clinical practice. Mobile health (mHealth) solutions are well‐positioned to help solve this issue, as they are widely accepted, accessible and inexpensive.^15^, ^17^, ^18^, ^19^ Additionally, mHealth has demonstrated its usefulness in several areas, including greater access to health information, remote patient monitoring and adherence measurement.^20^, ^21^, ^22^, ^23^, ^24^ In patients with asthma, smart device ownership levels are similar to those of the general population, with one‐third having used a health and fitness app and two‐thirds showing interest in using apps to support their asthma management.^25^

Previous studies have demonstrated the feasibility of using an asthma app to support medication management and adherence,^24^, ^26^ namely the InspirerMundi app. InspirerMundi was developed grounded in previous research and cooperation with patients with asthma and physicians.^27^, ^28^ The app aims to transform adherence to inhalers into a positive experience through gamification and social support while allowing for verified inhaler adherence monitoring through a medication detection tool based on advanced processing of inhaler images captured with the smartphone camera. A 1‐month mixed method multicenter observational study conducted has shown a median inhaler adherence of 62%.^24^ In this study's extension to 4 months, the median adherence fell to 34%, but asthma control scores improved during InspirerMundi use. Higher app use was associated with older age, taking medication for other health conditions and use of long‐acting muscarinic antagonists.^29^ Despite of these positive findings, none of these studies has compared adherence measured by the app with other measures currently used in clinical practice. However, in a clinical setting, any additional adherence measurement must be evaluated in the context of both the patient and physician perspectives so that it can also help improve the process of shared decision making.^30^ Thus, the purpose of this study is to compare methods to assess adherence to inhaled control medication in patients with asthma, and to the authors' knowledge, it is the first study comparing inhaler adherence measured through an asthma app, patient self‐report and physician assessment.

## 2 METHODS

### 2.1 Study design

This study is a secondary analysis of three prospective multicenter observational studies (mINSPIRE secondary care 2018–2019, mINSPIRE secondary care 2019–2020, mINSPIRE primary care 2020–2021) conducted with adolescents and adults with persistent asthma. The mINSPIRE studies are part of the Inspirers project, which is focused on improving the adherence to asthma inhalers in patients with persistent asthma.^31^ In the mINSPIRE studies, patients had an initial face ‐to‐ face visit in which they were invited to use the InspirerMundi app for 4 months. Patients were recruited through convenience sampling at 61 primary and secondary (allergy, pulmonology, and paediatric) care centres in Portugal and Spain, between March 2018 and October 2020.

The ethics committees of all participating centres approved the study protocol, and the study was conducted in agreement with the Declaration of Helsinki and the ethical standards thereby established. Written informed consent was obtained before enrolment in the study. In the case of adolescent patients, both signed assent and a parental consent form were obtained. The reporting of the study is done in accordance with STROBE (Strengthening the Reporting of Observational Studies in Epidemiology) guideline.^32^

### 2.2 Participants

The inclusion criteria in the mINSPIRE studies were (1) having a previous medical diagnosis of persistent asthma, (2) being at least 13 years old (13–17 years adolescents; ≥18 years adults), (3) having an active prescription for a daily inhaled controller medication for asthma, and (4) having access to a mobile device with a working Internet connection while being able to use mobile applications. No change was made to prescribed medication due to the study and all inhaled controller treatments were permitted. Additional inclusion criteria were considered for this secondary analysis: patients were required to have installed the InspirerMundi app and register at least one asthma control inhaler. The exclusion criteria consisted of (1) having a previous chronic condition that could interfere with the aims of this study, and (2) being diagnosed with a chronic lung disease other than asthma.

### 2.3 Data collection

App adherence rate was computed by the InspirerMundi app as the number of inhalations registered as taken each day divided by the number of planned (as prescribed) inhalations each day. The first week and first month marks were considered for analysis (1 week and 1 month, respectively). The main focus of this app is to promote patients' adherence to treatment and medication management. Therefore, the therapeutic plan agreed upon between patient and physician can be registered in the app, which in turn activates a reminder in the form of a notification when a medication is due. The inhaled intakes are then recorded through an image detection tool that relies on advanced image processing techniques and is enabled by the smartphone camera. This tool was specifically designed to support the objective assessment of adherence to inhaled medications using template matching to identify the inhaler^27^ and its dose counter. Throughout the studies, two versions (1.1 and 1.2) of the app were used, that supported the identification of, respectively, 6 and 12 inhaler devices with numeric dose counters. When the patient registers a medication intake of one of these inhalers, an initial screen with instructions on how to match the inhaler with the template is provided and the detection tool automatically confirms the inhaler. It is also possible to register inhalations from other inhalers. However, in these two cases the template matching feature is inactive. A scheduled intake was considered as taken when the inhaler is presented to the detection tool for 10 s. Moreover, the app can also track relief medication intake events, but these were not considered for evaluation of treatment adherence. In addition to these capabilities, other components are available, namely, adherence statistics in the form of circular progress graphs, as well as a timetable that represents the registered treatment plan. Both of these representations support medication management by providing the patient with a visual representation of their progress. Moreover, the user has the option to share the registered therapeutic plan and associated adherence data with their physician. A more detailed explanation of the different components of the app can be found in a previous study.^24^

During the initial in‐person visit, global adherence to asthma control inhalers during the previous week was assessed independently by both patients and physician using a visual analogue scale (VAS, 0—Worst; 100—Best).

Patients reported demographic data including age, gender, body mass index, smoking status. Current asthma treatment, biologic therapy, allergen immunotherapy and evaluation of asthma control was assessed by the physician according to the Global Initiative for Asthma (GINA).^1^ The use of health care resources in the former year was also recorded, namely the number of exacerbations (episodes of progressive increase in shortness of breath, cough, wheezing, and/or chest tightness, requiring change in maintenance therapy) and the number of unscheduled medical visits.

### 2.4 Data analysis

In the case of missing patient‐self report and physician assessment values, Little's test of missing completely at random (MCAR) was used. If the variables were indeed missing at random the imputation technique chosen was expectation maximisation as a comparison of imputation techniques for handling missing data found it produced estimates closest to those of the original variables.^33^ To characterise the sample, descriptive statistics were utilised. Visual analysis of histograms and Kolmogorov‐Smirnov tests were used to determine the normality of each variable. All inhaler adherence scores (app, patient, physician) were compared using the Friedman test and Wilcoxon signed‐rank tests, with Bonferroni correction being applied when conducting multiple comparisons.^34^ Spearman's rho was used to assess the correlation between all the adherence scores. Spearman's rho was interpreted as negligible (0–0.3), low (≥0.3–0.5), moderate (≥0.5–0.7), high (≥0.7–0.9) and very high (≥0.9–1) (26). Adherence scores were categorised using the clinically significant cut‐off of 75% (≤75%: non‐adherent; >75% adherent), as patients with asthma whose adherence was >75% have previously shown a significant decrease of exacerbations.^35^, ^36^ Weighted Cohen's kappa and percentage of agreement were used to determine the agreement of the adherence categories.^37^ Cohen's kappa values were interpreted as: <0, no agreement; 0–0.20, slight; 0.21–0.40, fair; 0.41–0.60, moderate; 0.61–0.80, substantial and 0.81–1.0, almost perfect agreement.^37^

In order to analyse the possible impact of the two app versions in the assessed inhaler adherence in the three studies, a Kruskal‐Wallis Test was used, followed by multiple comparisons using Mann‐Whitney *U* Test with Bonferroni correction.^34^ Mann‐Whitney *U* Test was also used to assess whether there was a difference in adherence between primary and secondary care. Statistical analyses were performed using IBM SPSS Statistics V.27.0 (IBM Corporation) and plots were created with GraphPad Prism V.9.0 (GraphPad Software). The significance level was set at 0.05.

## 3 RESULTS

### 3.1 Participants

**FIGURE 1:**
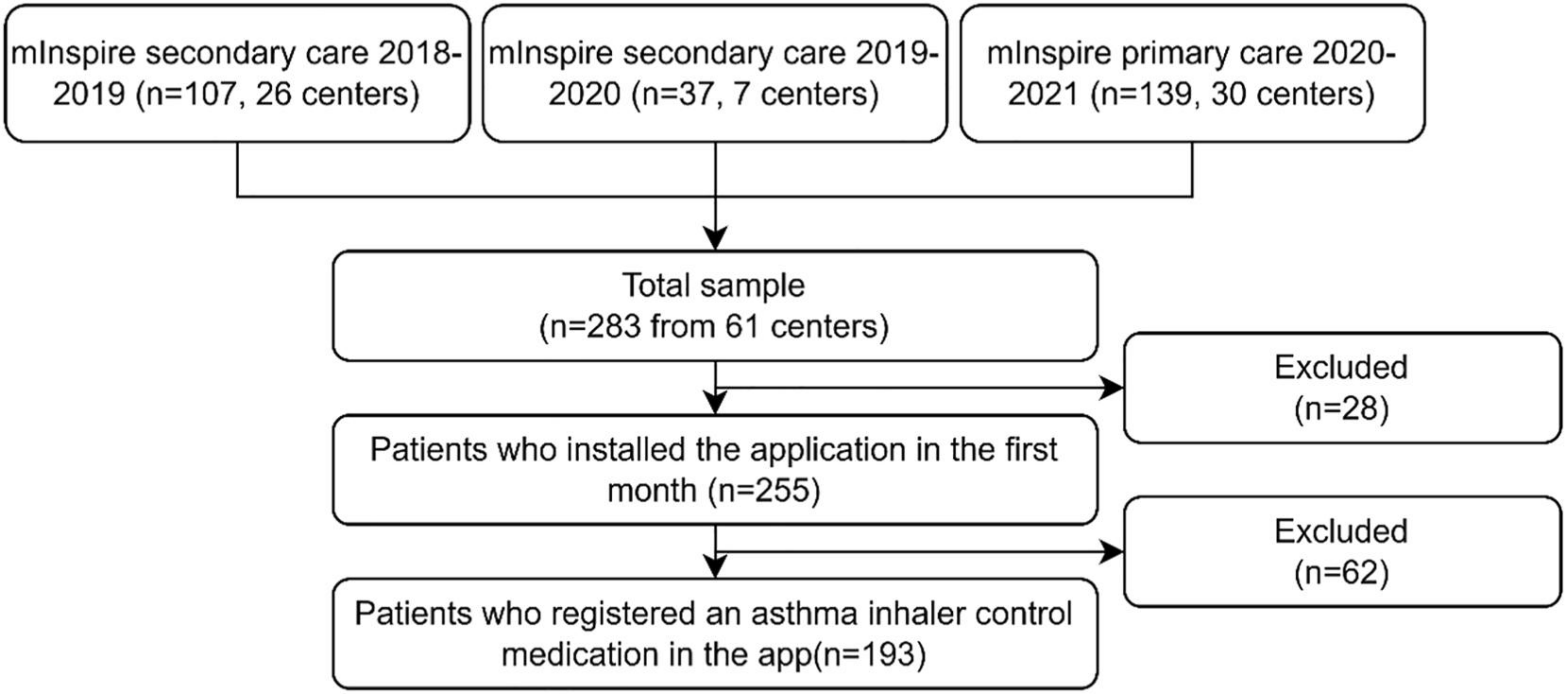
Patient flowchart of the secondary analysis conducted

From the 283 patients recruited, 193 (68%) registered at least one asthma inhaler control medication in the app and were analysed in this study (Figure 1). Patients had a median age (percentile 25 to percentile 75) of 28 (19–41) years and were mainly female (72%), see Table 1. Most were on inhaled corticosteroids (ICS)/long‐acting beta‐agonists combination therapy (*n* = 160; 83%) and used only one inhaler (*n* = 116; 60%). More than half of participants had their asthma not well‐controlled (*n* = 100; 56%) according to the GINA classification and nearly half had at least one exacerbation during the previous year (*n* = 79; 41%).

**TABLE 1:** Participants' baseline characteristics (*n* = 193)

	Total (*n* = 193)	mINSPIRE secondary care 2018–2019 (*n* = 68)	mINSPIRE secondary care 2019–2020 (*n* = 25)	mINSPIRE primary care 2020–2021 (*n* = 100)
Age, Mdn (P25–P75), years	28 (19–41)	23.5 (17–38.3)	22 (15–28)	33 (23–44.3)
Female	138 (72%)	42 (62%)	21 (84%)	75 (75%)
BMI, Mdn (P25–P75), kg/m^2^	23.2 (21.2–26.8)	22.8 (21.2–25.2)	22.5 (21.1–24.1)	24.2 (21.3–27.8)
Smoking				
Never	140 (73%)	51 (75%)	22 (88%)	67 (67%)
Ex	36 (19%)	4 (6%)	3 (12%)	23 (23%)
Current	13 (7%)	13 (19%)	0 (0%)	0 (0%)
Inhaled medication				
ICS‐LABA	160 (83%)	42 (62%)	23 (92%)	95 (95%)
ICS	18 (9%)	9 (13%)	2 (8%)	7 (7%)
LAMA	14 (7%)	8 (12%)	2 (8%)	4 (4%)
SABA	51 (26%)	10 (15%)	13 (52%)	28 (28%)
Single inhaler	116 (60%)	44 (65%)	11 (44%)	61 (61%)
Oral medication anti‐leukotrienes	75 (39%)	31 (46%)	13 (52%)	31 (31%)
Allergen immunotherapy	25 (13%)	12 (18%)	3 (12%)	10 (10%)
Biologic therapy	12 (6%)	7 (10%)	2 (8%)	3 (3%)
GINA assessment symptom control				
Well‐controlled	84 (44%)	34 (50%)	11 (44%)	39 (39%)
Partly controlled/uncontrolled	108 (56%)	34 (50%)	14 (56%)	60 (60%)
≥1 exacerbation past year	79 (41%)	29 (43%)	13 (52%)	37 (37%)
≥1 unscheduled medical visit past year	42 (22%)	14 (21%)	8 (32%)	20 (20%)

*Note*: Values are shown as *n* (%) unless otherwise indicated.

Abbreviations: BMI, body mass index; GINA, Global Initiative for Asthma; ICS, inhaled corticosteroids; LABA, long‐acting beta‐agonists; LAMA, long‐acting muscarinic receptor antagonists; Mdn, median; P25–P75, percentile 25 to percentile 75; SABA, short‐acting beta‐agonists.

### 3.2 Inhaler adherence

**FIGURE 2:**
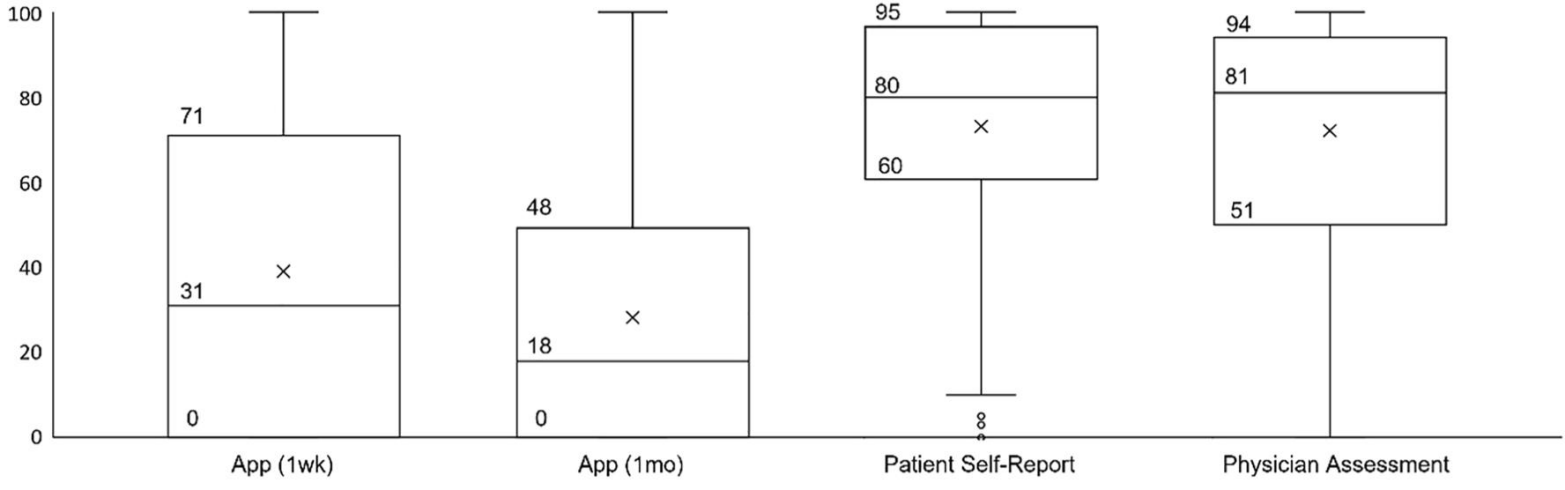
Box plots showing app‐measured adherence rates (1 week and 1 month), patient self‐report, and physician assessment of adherence, median (percentile 25–percentile 75). App‐measured adherence rate 1 week: 31 (0–71); App‐measured adherence rate 1 month: 18 (0–48); Patient self‐report: 80 (60–95); physician assessment: 81 (51–94); The mean adherence in each case is indicated by an *x*. *Y* axis is the adherence value.

The percentage of missing values across patient self‐report and physician assessment varied between 0% and 5%. In total, 12 patients' records (6%) were incomplete. A Missing Values Analysis indicated that Little's test of MCAR was not significant (*χ* ^2^ = 24.475, DF = 29, *p* = 0.705). Adherence measures did not follow a normal distribution (1 week: *D* = 0.174, *p* < 0.001; 1 month: *D* = 0.175, *p* < 0.001; Patient: *D* = 0.1192; *p* < 0.001; Physician: *D* = 0.215, *p* < 0.001). Adherence measurements had significantly different distributions (*χ* ^2^ = 220.658, DF = 3, *p* < 0.001) (Figure 2). On average, app‐measured adherence rate was lower (1 week: Mdn = 31%, P25–P75 = 0–71; 1 month: Mdn = 18%, P25–P75 = 0–48) than patient self‐report and physician assessment (Patient: Mdn = 82; Physician: Mdn = 82), and this difference was statistically significant (*p* < 0.001). There was not a significant difference between the distribution of patient and physician data (*p* = 0.828).

**FIGURE 3:**
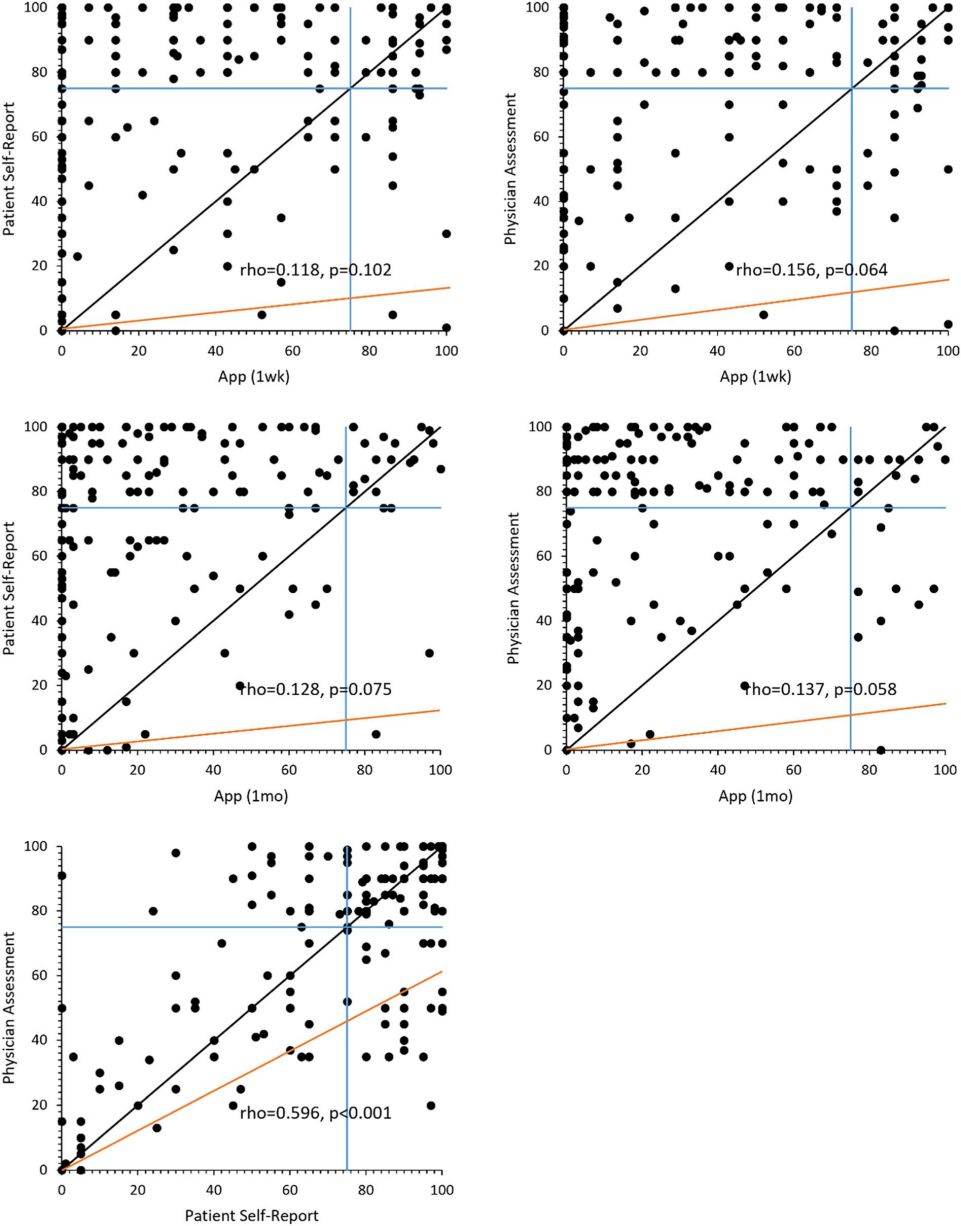
Scatter plots showing the relationship between application, physicians, and patients estimates of inhaler adherence. Orange line showing correlation, blue lines showing 75% cut‐offs and black line showing perfect agreement.

Correlations between app‐measured adherence rates and patient self‐report and physician assessment were non‐significant, while there was a significant low to moderate correlation between patient and physician (*ρ* = 0.596, *p* < 0.001; Figure 3). According to app data, 22% of patients were classified as adherent in the first week and 13% in the first month; patients self‐report indicated 60% of patients as being adherent and physician assessment indicated 62%. Based on category discordance, a weighted Cohen's kappa was found not to be significant between the app‐measured adherence rate and both patient and physician evaluation, with the exception being the app‐measured adherence rate in the first month and patient self‐report which had slight agreement (app 1 week and patient: *κ* = 0.036, *p* = 0.494; app 1 week and physician: *κ* = 0.087, *p* = 0.084; app 1 month and Patient: *κ* = 0.105, *p* = 0.010; app 1 month and physician: *κ* = 0.003, *p* = 0.930). Physicians overestimated adherence in 45% of cases compared with the app at 1 week and 55% at 1 month, while underestimating in 6% at 1 week and 4% at 1 month. Patients in the same way overestimated adherence in 47% of cases at 1 week and 54% at 1 month, while underestimating in 5% at 1 week and 1% at 1 month. The weighted Cohen's kappa between patient and physician revealed moderate agreement (*κ* = 0.462, *p* < 0.001).

Significant differences on app‐measured adherence rate were found among the three studies (1 week: *H* = 21.499, *p* < 0.001, df = 2; 1 month: *H* = 24.333, *p* < 0.001, df = 2), with a statistically significant higher app‐measured adherence rate in the mINSPIRE 2018–2019 study (1 week: Mdn = 69%; 1 month: 36%) than in 2019–2020 (1 week: Mdn = 7%; 1 month: Mdn = 2%) and 2020–2021 (1 week: Mdn = 21%; Mdn = 9%) studies. App‐measured adherence rate was higher in secondary care (1 week: Mdn = 57%; 1 month: 23%) than in primary care (1 week: Mdn = 23%; 1 month: 9%; 1 week: *U* = 5858, *p* = 0.002; 1 month: *U* = 5802, *p* = 0.003).

## 4 DISCUSSION

Our results show that adherence as measured by the application was lower than that reported by the patient or the physician. Furthermore, there was no relevant correlation between app‐measured adherence rate and the subjective measurements.

The disparity between subjective and objective measurements was expected, as objective measurements of adherence values are commonly lower than those of subjective evaluations.^13^, ^38^ There was a correlation between patient's self‐report and physician assessment which was consistent with previous studies in the same line of research.^16^ However, patients and physicians still discord in half of the cases which illustrates the need for the integration of objective adherence measures.^16^, ^39^ Both the percentage of patients classified as adherent (22% 1 week; 13% 1 month) and the adherence rate as measured through the app (Mdn 31% 1 week; 18% 1 month) was similar to other objective measurements reported in a recent meta‐analysis of adherence to ICS in young adults (15–30 years).^38^ In this meta‐analysis, the percentage of adherent patients as measured through pharmacy refill was 20% and follow‐up times ranged from 1 week to several months. The only two studies of the meta‐analysis with electronic monitoring devices had an adherence rate of 12.5% and 35%, with one having a duration of 3 months and the other not specifying the duration.^38^, ^40^ However, it is important to note that the difference in follow‐up times could limit direct comparisons with the present study. Moreover, subjective measurements also had the highest adherence estimates in both the meta‐analysis (35% patients classified as adherent), and the three mINSPIRE studies (60% classified as adherent). There were, however, patients in which app adherence was higher than that of the physician assessment and self‐report, which could be explained by their participation in the study.

It is also important to notice that the adherence rate also decreased significantly from the first week to the first month, a pattern observed in previous studies^41^, ^42^ but also in real‐world clinical practice. This can be more worrisome if we consider that this trend was observed in patients that accepted to use the app and participate in the study and thus could be worse for real‐world patients not evaluated in the study. Nevertheless, we also need to consider, that participants were not selected based on their interest on the app social and gaming components and it is possible that in patients with higher interest in these features, app adherence would be higher.^43^ Moreover, even in the patients who indicated their planned inhalations in the app it is possible that the lower adherence values of the app when compared with the patient and physician values could be due to the patients not interacting with the app when taking their medication, this is an important factor and one that needs to be considered in future analysis. The improvement of the app, namely through the real‐time inhaler dosage counter value identification module will be a major step forward.^28^ This module uses the smartphone camera and provides, through image processing techniques and machine learning tools, to confirm the inhaler presented to the camera and the dosage value inferred from the acquired dose counter image.^28^ This will allow a more objective verification of inhaler adherence, as based on the therapeutic plan registered and on dose counter read, the app will be able to check missed doses and calculate real adherence, even when app is not used in a daily basis. Moreover, improvements in the app regarding gamification and social interaction will also further promote medication adherence.^44^ Yet app improvements will not replace the importance of providing additional interventions and follow‐ups at later time points as opportunities to increase adherence and help improve patient monitoring.^45^ These could have multiple components, including educational interventions, as patients with sufficient knowledge about asthma medication are significantly more likely to have better adherence.^46^

App adherence rate was higher in 2018–2019 when compared with the latter two studies (2019–2020 and 2020–2021). No explanation was found to explain the difference between the 2018–2019 and 2019–2020 study. However, possible causes for the higher adherence in the 2018–2019 study include an older sample and a higher proportion of participants on a single inhaler, both factors associated with higher adherence.^38^ Furthermore, app‐measured adherence was significantly higher in secondary care when compared with primary care. This could be due to patient in secondary care being typically more symptomatic and subsequently more motivated to adhere to therapy in the hope of reducing the symptoms.^47^, ^48^ In addition, secondary care provides more opportunities for educating the patient, with physicians who are more likely to have the resources to provide this information.^38^, ^49^ Another factor that could have influenced the 2020–2021 results was the COVID‐19 pandemic. It is unclear however whether asthma inhaler adherence improved or decreased during this period, as there are contradictory findings.^50^, ^51^

The inclusion of both primary and secondary care patients is one of the strengths of this study, with patients being recruited from 61 care centres, in addition to having broad age range. This is particularly important because, starting in adolescence, patients instead of their parents become responsible for the majority of daily controller‐medication.^52^ Possible limitations include the recruitment process, which was done by convenience sampling, including only patients who had persistent asthma and only measuring the patient and physician perspectives at baseline. This could limit the generalisation of these findings to all asthma patients and future studies should, if possible, include consecutive or random sampling, and a greater duration of follow‐up. Despite adherence measured by the app was similar to other objective measurements reported in the literature, we cannot exclude that under‐ or overestimation by the app is still possible. In the versions of the app used in those studies it was not yet possible to read and confirm the number in the inhaler dose counters, and inhaler doses were counted as taken based on the inhaler template matching feature. Future studies using the real‐time inhaler dosage counter value identification module will clarify this. In addition, the comparison with other objective measures of inhaler adherence, such as pharmacy refill data, was not possible. Nevertheless, we need to consider that currently available objective methods require laborious analysis by physicians, are costly to implement in clinical practice and, like inhaled corticosteroid serum level and FeNO suppression test,^53^, ^54^ are not fully validated. Another important limitation is that patient and physician adherence data was retrieved at a different time point than that of the application, therefore a future improvement would be tracking all measurements at the same time points. Moreover, a previous study found that asthma control and FEV1 were significantly associated with patient‐physician disagreement in what regards inhaler adherence.^16^ However, no app data was included in that previous work. Futures studies could further explore the factors associated with the disagreement between different measures of inhaler adherence. Despite these acknowledge limitations, since there are no ideal measurements when it comes to adherence, we believe this paper is a necessary starting point to better understand how different ways of measuring adherence relate to each other. These results will be fundamental to improve future studies exploring the impact of adherence on asthma control and other disease outcomes.

In conclusion, adherence measured by the app was lower than that reported by the patient or the physician. This was expected as objective measurements are commonly lower than subjective evaluations, which tend to overestimate adherence. Nevertheless, the low adherence measured by the app may also be influenced by the use of the app itself and this needs to be considered in future studies.

## AUTHOR CONTRIBUTIONS

Conceptualization: Afonso Cachim, Cristina Jácome, João Almeida Fonseca. Data curation: Afonso Cachim, Cristina Jácome, Rita Amaral. Methodology: Afonso Cachim, Cristina Jácome, Rita Amaral, Ana Margarida Pereira, Rute Almeida and João Almeida Fonseca. Writing—original draft: Afonso Cachim. Writing—review and editing: Afonso Cachim, Ana Margarida Pereira, Rute Almeida, Rita Amaral, Magna Alves‐Correia, Pedro Vieira‐Marques, Claudia Chaves‐Loureiro, Carmelita Ribeiro, Francisca Cardia, Joana Gomes, Carmen Vidal, Eurico Silva, Sara Rocha, Diana Rocha, Maria Luís Marques, Rosália Páscoa, Daniela Morais, Ana Margarida Cruz, Marta Santalha, José Augusto Simões, Sofia da Silva, Diana Silva, Rita Gerardo, Filipa Todo Bom, Ana Morete, Inês Vieira, Pedro Vieira, Rosário Monteiro, Maria Rosário Raimundo, Luís Monteiro, Ângela Neves, Carlos Santos, Ana Margarida Penas, Rita Regadas, José Varanda Marques, Inês Rosendo, Margarida Abreu Aguiar, Sara Fernandes, Carlos Seiça Cardoso, Filipa Pimenta, Patrícia Meireles, Mariana Gonçalves, João Almeida Fonseca, Cristina Jácome. All authors have read and agreed to the published version of the manuscript.

## CONFLICT OF INTEREST

JAF is the co‐founder of SME, which owns the InspirerMundi app. The authors declare that they have no competing interests.

## CONSENT TO PARTICIPATE

The study was conducted according to the guidelines of the Declaration of Helsinki and approved by the ethics committees of all participating centres.

## CONSENT FOR PUBLICATION

Not applicable.
